# Disaggregating Tropical Disease Prevalence by Climatic and Vegetative Zones within Tropical West Africa

**DOI:** 10.1371/journal.pone.0152560

**Published:** 2016-03-29

**Authors:** Carl S. Beckley, Salisu Shaban, Guy H. Palmer, Andrew T. Hudak, Susan M. Noh, James E. Futse

**Affiliations:** 1 Department of Animal Science, College of Basic and Applied Sciences, University of Ghana, Legon, Ghana; 2 Animal Research Institute, The Council for Scientific and Industrial Research (CSIR), Frafraha, Accra, Ghana; 3 Animal Disease Research Unit, Agricultural Research Service, U.S. Department of Agriculture, Pullman, WA, United States of America; 4 Program in Vector-Borne Diseases, Department of Veterinary Microbiology and Pathology, Washington State University, Pullman, WA, United States of America; 5 Paul G. Allen School for Global Animal Health, Washington State University, Pullman, WA, United States of America; 6 Rocky Mountain Research Station, Forest Service, U.S. Department of Agriculture, Moscow, ID, United States of America; Institut Pasteur, FRANCE

## Abstract

Tropical infectious disease prevalence is dependent on many socio-cultural determinants. However, rainfall and temperature frequently underlie overall prevalence, particularly for vector-borne diseases. As a result these diseases have increased prevalence in tropical as compared to temperate regions. Specific to tropical Africa, the tendency to incorrectly infer that tropical diseases are uniformly prevalent has been partially overcome with solid epidemiologic data. This finer resolution data is important in multiple contexts, including understanding risk, predictive value in disease diagnosis, and population immunity. We hypothesized that within the context of a tropical climate, vector-borne pathogen prevalence would significantly differ according to zonal differences in rainfall, temperature, relative humidity and vegetation condition. We then determined if these environmental data were predictive of pathogen prevalence. First we determined the prevalence of three major pathogens of cattle, *Anaplasma marginale*, *Babesia bigemina* and *Theileria* spp, in the three vegetation zones where cattle are predominantly raised in Ghana: Guinea savannah, semi-deciduous forest, and coastal savannah. The prevalence of *A*. *marginale* was 63%, 26% for *Theileria* spp and 2% for *B*. *bigemina*. *A*. *marginale* and *Theileria* spp. were significantly more prevalent in the coastal savannah as compared to either the Guinea savanna or the semi-deciduous forest, supporting acceptance of the first hypothesis. To test the predictive power of environmental variables, the data over a three year period were considered in best subsets multiple linear regression models predicting prevalence of each pathogen. Corrected Akaike Information Criteria (AICc) were assigned to the alternative models to compare their utility. Competitive models for each response were averaged using AICc weights. Rainfall was most predictive of pathogen prevalence, and EVI also contributed to *A*. *marginale* and *B*. *bigemina* prevalence. These findings support the utility of environmental data for understanding vector-borne disease epidemiology on a regional level within a tropical environment.

## Introduction

The World Health Organization defines tropical diseases as encompassing “all diseases that occur solely, or principally, in the tropics” and that “in practice, the term is often taken to refer to infectious diseases that thrive in hot, humid conditions” (http://www.who.int/topics/tropical_diseases/en/) [1]. While tropical infectious disease prevalence is, in general, dependent on a broad number of factors, including economic, demographic, and socio-cultural determinants, rainfall and temperature frequently underlie overall prevalence [2, 3]. This is especially true for arthropod vector-borne diseases for which vector presence, abundance, activity, and seasonality are highly dependent on climate [4–6]. As a result vector-borne diseases, including “targeted” diseases such as malaria as well as neglected infectious diseases, have a highly skewed distribution with increased prevalence in tropical countries [7–10].

Specific to Africa, the tendency to incorrectly infer that tropical diseases are uniformly prevalent throughout the roughly 75% of the continent that lies within the tropics has been overcome, at least partially, with solid epidemiologic data, including the data presented here from Ghana [11]. This finer resolution epidemiologic data has at least two important implications. First is that prevalence data can guide treatment, especially in areas where the diagnosis is primarily based on clinical signs [12–14]. Illustrative of this are findings from northern Tanzania in which non-malarial febrile illness greatly exceeded the proportion attributed to malaria and for which different therapy is required [15]. Second is the importance for population immunity. Boundaries where higher prevalence zones, with a correspondingly higher level of population immunity, intersect with zones of lower prevalence and low population immunity create risk for more rapid spread and more severe disease if the underlying transmission determinants change.

**Fig 1:**
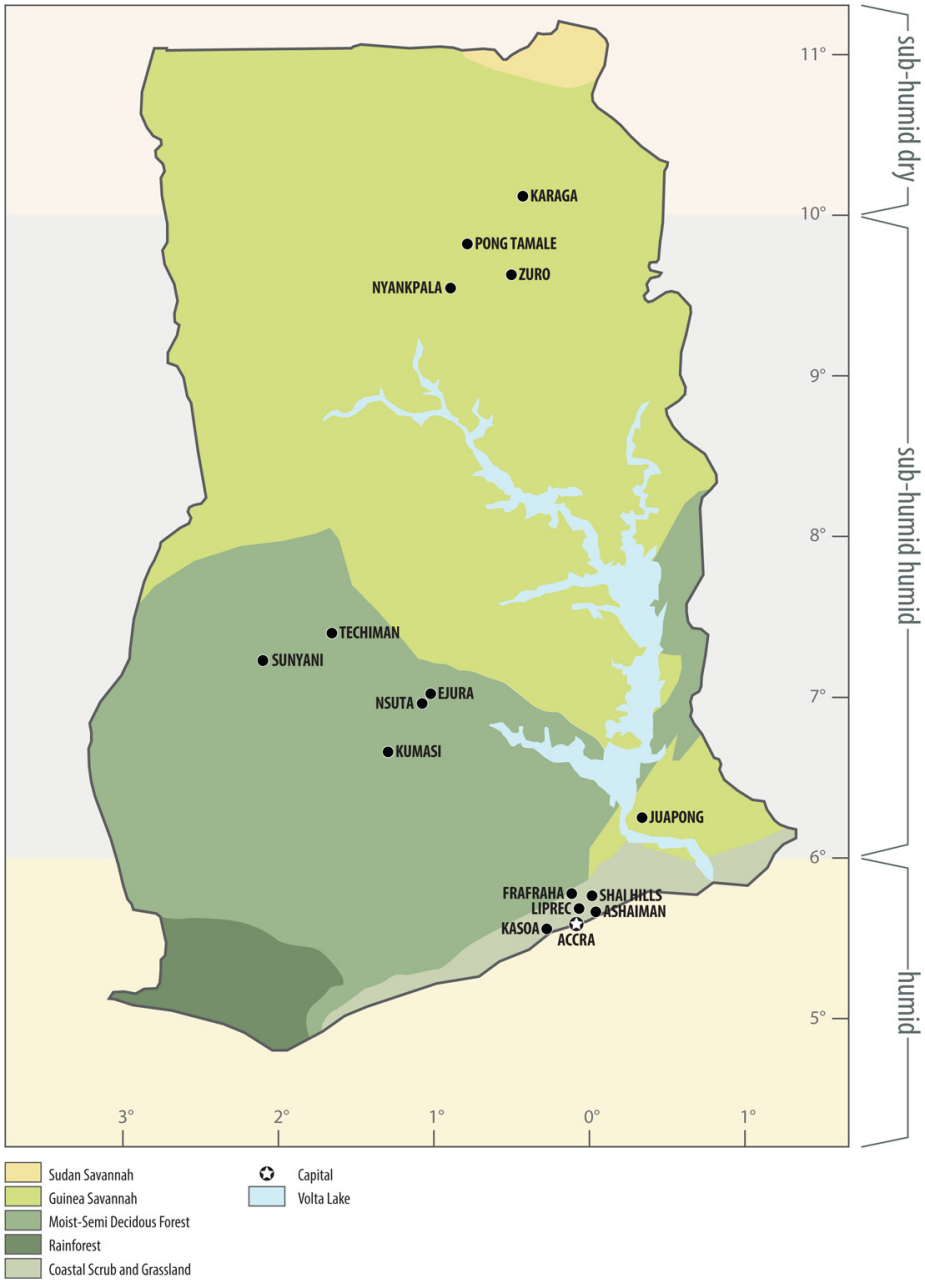
Sampling locations within three vegetation zones in Ghana. Samples from cattle were collected from multiple sites per vegetation zone, marked with black dots. EVI was extracted from MODIS satellite imagery from the same sites. The capital of Ghana, Accra, is marked with a star.

We hypothesized that vector-borne pathogen prevalence would significantly differ according to zonal differences in environmental parameters such as rainfall, temperature, relative humidity and vegetation, even in the overall context of a national tropical climate. We addressed this question by determining the prevalence of tick-borne pathogens within three vegetation zones of Ghana (Fig 1). These areas are located entirely within the tropics (between 4° and 12°N, 4°W and 2°E) and are considered to have a tropical climate at the national level as characterized by high mean temperature and rainfall. Despite this national level tropical classification, Ghana has three climatic zones (humid, sub-humid humid, and sub-humid dry) and encompasses a variety of vegetation zones (rain forest, semi-deciduous forest, Guinea savannah, Sudan savannah and coastal savannah). To control for movement between climatic regions, we determined the prevalence of tick-borne pathogens in cattle raised exclusively within the three distinct vegetation zones in which cattle are predominantly raised: the Guinea savannah, the semi-deciduous forest and the coastal savannah. Here, we report the testing of the hypothesis of significant differences in pathogen prevalence in cattle within a national level tropical climate, determine if weather data and the enhanced vegetation index (EVI) could be used to predict pathogen prevalence and discuss the results in the context of transmission and mitigation of disease risk.

## Materials and Methods

### Zonal climatic characteristics

The monthly means of the minimum and maximum temperature, relative humidity and rainfall (mm) were obtained from Ghana Meteorological Authority for 2010, 2011, and 2012 for the Guinea savannah (Tamale), the deciduous forest zone (Sunyani), and the coastal savannah (Accra region). Statistically significant differences in minimum and maximum temperatures, relative humidity and rainfall among the three zones were determined using an ANOVA followed by Tukey’s HSD for pairwise comparisons and JMP software version 10.0.0 (SAS Institute Inc., Cary NC). For each variable (temperature, relative humidity and rainfall), zone, year, month and all interacting effects were tested. Also considered as a predictor variable was the Enhanced Vegetation Index (EVI) calculated from Moderate Resolution Imaging Spectroradiometer data (MODIS), which are collected daily and globally at 250 meters spatial resolution and composited every 16 days into the MOD13Q1 data product. The compositing process effectively reduces pixel contamination by clouds or cloud shadows. The EVI is designed to provide consistent spatial and temporal comparisons of vegetation conditions. The USGS MODIS Re-projection Tool Web Interface was used to generate downloadable Geo-Tiff files of the MOD13Q1 imagery, from which EVI was extracted at the 15 study sites. Satellite and weather data were entered into a spreadsheet and exported for analysis to the *R* platform for computing [16].

### Determination of infection prevalence

*Anaplasma marginale*, *Babesia bigemina*, and *Theileria* spp. have previously been identified in cattle on a national scale within Ghana and have been targeted as pathogens that constrain livestock production [17]. Blood samples were collected from animals in each of the three targeted climatic zones and included 131 animals in Guinea savannah (5 sites), 69 animals in the deciduous forest (5 sites), and 197 animals in the coastal savannah (5 sites) collected between 2010 and 2012. Genomic DNA was then extracted (Qiagen) and a previously validated multiplex PCR used to determine infection prevalence [18]. Briefly, the primers used were specific for a 265 bp fragment of *A*. *marginale msp1β* (forward, 5’ gctctagcaggttatgcgtc 3’; reverse 5’ ctgcttgggagaatgcacct 3’); an 1125 bp fragment of *B*. *bigemina cytochrome b oxidase* (forward, 5’ tggcggcgtttattagttcg 3’; reverse 5’ ccacgcttgaagcacagga 3’); and a 462 bp fragment common to the *cytochrome b oxidase* in multiple *Theileria* spp. (forward 5’ actttggccgtaatgttaaac 3’; reverse 5’ ctctggaccaactgtttgg 3’). Amplification conditions were an initial denaturation step of 95°C for 5 min, followed by 30 cycles of 94°C for 5 sec, 55°C for 30 sec, and the initial extension at 72°C for 45 sec. The products were finally extended at 72°C for 7 min before holding at 10°C. The minimum detection limits of pathogen DNA were 0.1ng, 10ng and 50ng using pathogen-specific primers for *A*. *marginale*, *Theileria spp*. and *Babesia spp*., respectively. In order to confirm the reported levels of sensitivity, these standards were included in each PCR assay.

### Relationship of infection prevalence to environmental data

**Fig 2:**
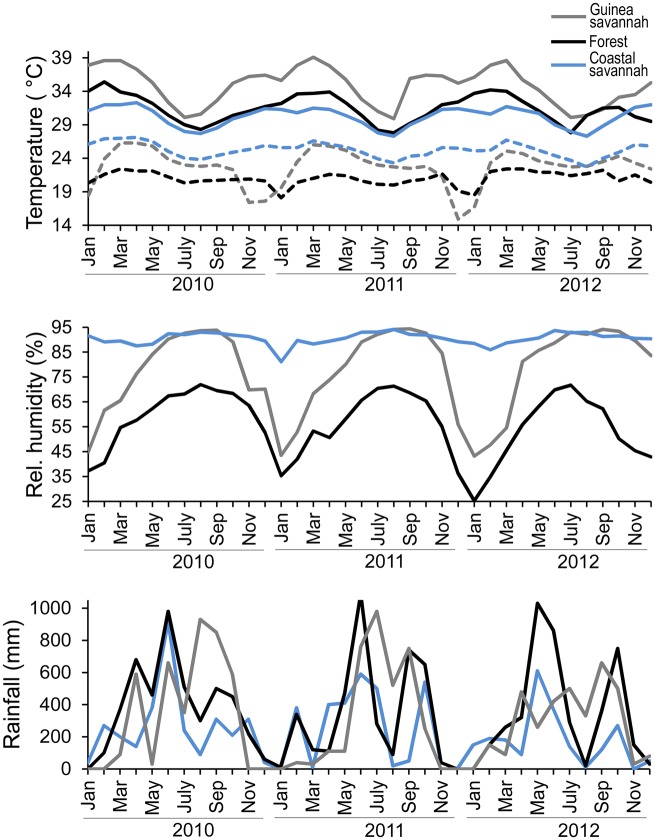
Monthly minimum and maximum temperatures, relative humidity and rainfall in the three zones of interest. a) Minimum (dashed lines) and maximum temperatures (solid line) for the Guinea savannah, deciduous forest, and coastal savannah from 2010 through 2012. b) Percent relative humidity for the Guinea savannah, deciduous forest and coastal savannah from 2010 through 2012. c) Rainfall for the Guinea savannah, deciduous forest, and coastal savannah from 2010 through 2012. These variables are represented by a single weather station in each zone.

**Fig 3:**
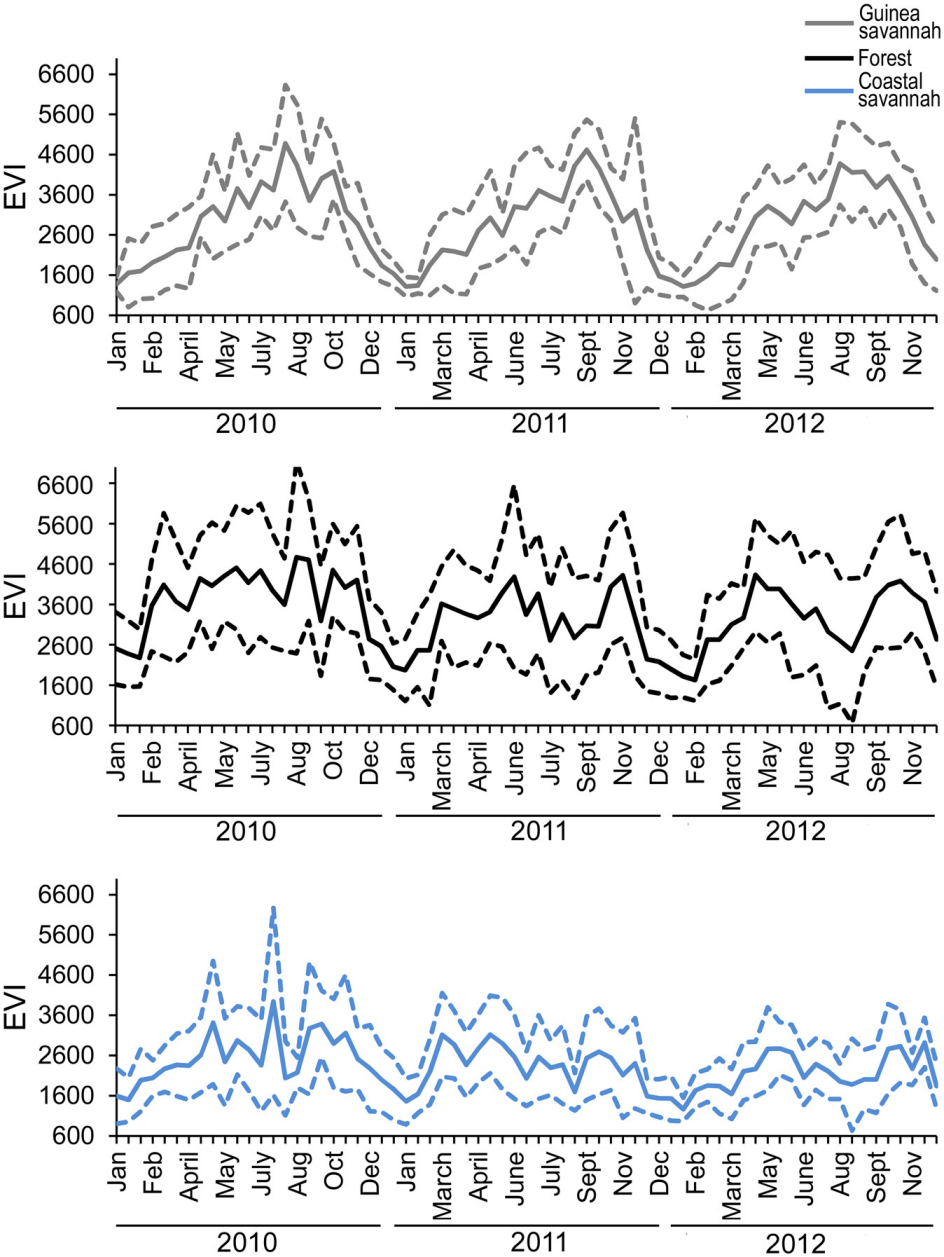
Enhanced Vegetation Index (EVI). EVI from 2010 through 2012 in the a) Guinea savannah, b) deciduous forest, and c) coastal savannah. EVI was extracted from MODIS satellite imagery at five sites per zone, from which mean (solid line) and +/- 1 standard deviation (dashed lines) statistics were derived.

Because infection prevalence was not temporally explicit within the 3-year period of interest for the 15 sites, the 3-year weather and EVI records (Figs 2 and 3) were averaged at each site and tested as candidate predictors of infection prevalence using ordinary least squares multiple linear regression. A best subsets procedure was applied using the ‘leaps’ package in R [16] to exhaustively search for the best combinations of environmental variables to use in the most predictive yet parsimonious models, as determined using the Akaike information criterion corrected (AICc) for small sample size. Model residuals were normal and without spatial autocorrelation based on the Shapiro-Wilk test [19] and Moran’s I statistic [20]. To compare the relative quality of the alternative regression models, the change in the corrected Akaike information criterion (delta AICc) between the best and competing models was calculated and Akaike weights assigned [21] using the ‘AICcmodavg’ package in R [16]. Models with delta AICc of 0–2 have substantial support; models with delta AICc of 4–7 have considerably less support; models with delta AICc >10 have essentially no support [21]. Evidence ratios for comparing competing models were calculated by dividing the AICc Wt of the best model by the AICc Wt of a competing model.

### Determination of transmission pressure

To test whether transmission was ongoing, as opposed to a single episodic event, blood samples were collected from 19 animals at LIPREC biweekly over a 12 week period (July to October 2013) after the initial sampling and then biweekly for an additional 24 weeks from the whole herds at Shai Hills, LIPREC and Ashiaman during March to September 2014, which is primarily during the rainy season when transmission is expected to be the greatest. Genomic DNA was extracted (Qiagen) and multiplex PCR performed as described and referenced above.

### Ethics statement

The cattle used in this study from all locations were treated in strict accordance to guidelines set by University of Ghana Institutional Animal Care and Use Committee.

The protocol was approved, for use in sampling blood from cattle in all locations within Ghana, by the Noguchi Memorial Institute for Medical Research’s (NIACUC protocol number: 2015-01-5X).

## Results

### Zonal characteristics

Maximum temperatures in all three zones exceeded 30°C with seasonal variation in the Guinea savannah and the deciduous forest zones (Fig 2). Similarly, relative humidity varied dramatically by season in these two zones, from <50 to >90% and 35–70% in the Guinea savannah and the deciduous forest zone, respectively. In contrast, temperature and especially relative humidity varied across a much smaller range within the coastal savannah (Fig 2). There were statistically significant differences in maximum temperature, minimum temperature, and relative humidity (p < 0.0001) with each zone being significantly different from the other two zones. In the case of rainfall (Fig 2), there were also statistically significant differences between the zones (p = 0.0063), with the deciduous forest zone being statistically significantly different than the coastal savannah; though the Guinea savannah was not statistically significantly different from either the deciduous forest zone or the coastal savannah. Only zone, month and the interacting effect of zone*month were statistically significant in all cases and thus were the only effects included in the final statistical analysis. Rainfall was episodic in all three zones while the EVI analysis revealed substantial seasonal changes in vegetation in the Guinea Savannah relative to the deciduous forest and the coastal savannah (Fig 3).

### Prevalence of tick-borne pathogens by vegetation zone

**Fig 4:**
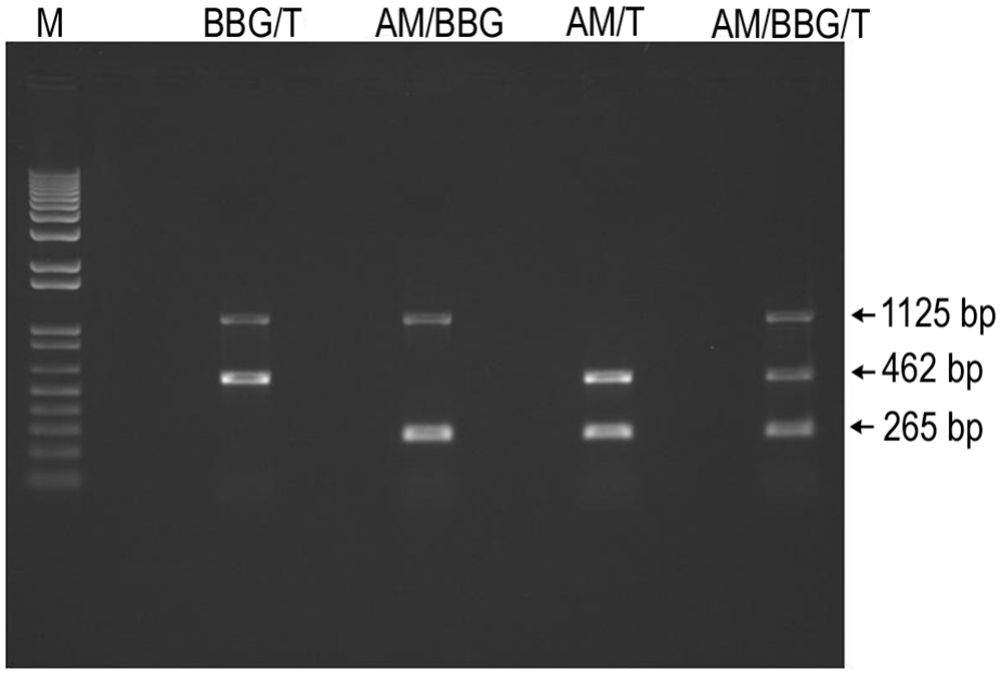
Detection of targeted pathogens by multiplex PCR. M represents the 1Kb plus DNA ladder. BBG+T represents PCR amplicons from simultaneous amplification of *B*. *bigemina* and *Theileria species*. AM+BBG represents PCR amplicons from simultaneous amplification of *A*. *marginale* and *B*. *bigemina*. AM+T PCR represents amplicons from simultaneous amplification of *A*. *marginale* and *Theileria species*. AM+BBG+T represents PCR amplicons from simultaneous amplification of *A*. *marginale*, *B*. *bigemina* and *Theileria species*.

All three targeted pathogens could be detected by multiplex PCR, either individually or as co-infections (Fig 4). The level of sensitivity for each pathogen was confirmed using standards in each assay. A total of 397 cattle were sampled from 15 locations (131 from the Guinea savannah, 69 and 197 from the semi-deciduous forest and coastal savannah, respectively). Of the three targeted tick-borne pathogens, *A*. *marginale* was the most prevalent in all three zones (45–75%) followed by *Theileria* spp. (13–34%) (Table 1). *B*. *bigemina* was least prevalent (3%) and the few infected animals were all co-infected with *A*. *marginale*. By vegetation zone, vector-borne infections were significantly more prevalent in the coastal savannah as compared to either the Guinea savannah or the deciduous forest (p = 0.001; Chi-Square Goodness-of-Fit test). This held true for both *A*. *marginale* and *Theileria* spp. as individual pathogens (p = 0.001) and as co-infections (p = 0.001).

**Table 1:** Prevalence of targeted pathogens by vegetative zone.

Vegetative zones	*A*. *marginale*	*B*. *bigemina*	*Theileria* spp.	Multiple pathogens^a.^
Guinea savannah	71/131 (54%)	4/131 (3%)	25/131 (19%)	20/131 (15%)
Deciduous forest	31/69 (45%)	0/69 (0%)	9/69 (13%)	10/69 (15%)
Coastal savannah	148/197 (75%)	6/197 (3%)	68/197 (34%)	75/197 (38%)
Total	250/397 (63%)	10/397 (2%)	102/397 (26%)	105/397 (26%)
χ^2^ (2df, α = 0.05)	85.8	5.7	55.3	70.7
p value	0.001 ^b.^	0.059	0.001 ^b.^	0.001 ^b.^

^a.^ Animals infected with more than one pathogen.

^b.^ Coastal savannah has significantly higher prevalence as compared to the Guinea savannah and deciduous forest zones.

### Prevalence of tick-borne pathogens by breed

The four most common breeds of cattle [22] were sampled and included Gudali (n = 76), Sanga (n = 151), West African Shorthorn (WASH; n = 113), and White Fulani (n = 57). We next determined if the difference in the distribution of cattle breeds among the zones or if differences in breed susceptibility could account for the differences in pathogen prevalence among the climate zones. There was no significant difference in breed distribution among the three climatic zones (p>0.05) nor was there a significant difference in overall infection prevalence or the prevalence of co-infection when comparing the different breeds (p>0.05).

### Evidence for continuous transmission within the coastal savannah

**Fig 5:**
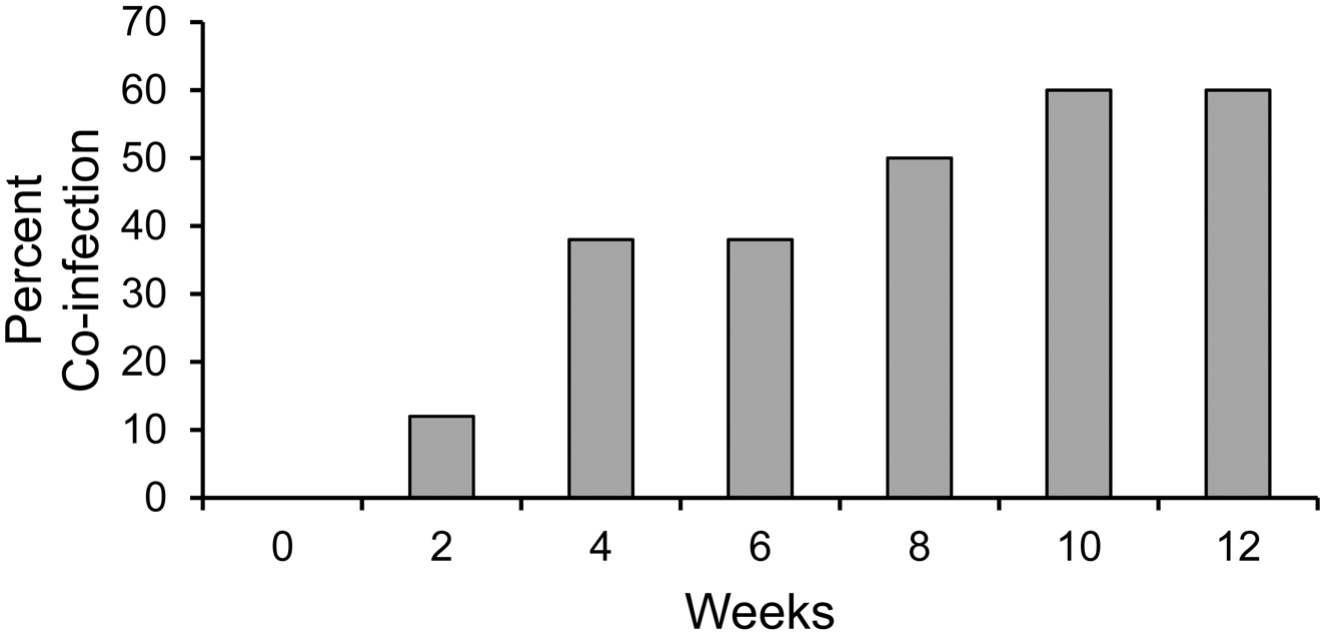
The percentage of animals acquiring more than one pathogen within a twelve-week period. The x-axis represents the percentage of animals infected with more than one pathogen. The y-axis represents the time of sampling in weeks.

The prevalence of animals infected with more than one of the targeted pathogens was highest in the coastal savannah with 38% of animals infected with more than one pathogen. In the Guinea savannah and forest regions, 15% of cattle had multiple infections. Based on this higher pathogen prevalence in the coastal savannah, we hypothesized that pathogen transmission pressure was intense. To test this hypothesis, we selected individual animals that were known to harbor a single pathogen and then determined the rate of infection with a second pathogen. Initially, 19 animals infected with a single pathogen were examined at bi-weekly interval for 12 additional weeks; 12% of these animals acquired a second pathogen within the first two-week interval (Fig 5). Over 50% of these animals became infected with one or more pathogen during the first eight weeks of observation. This increased to 60% and remained stable in weeks 10 and 12.

To determine if this high intensity transmission was mirrored on a larger scale, the rate of acquisition of a second pathogen was determined for three different herds, located 8 miles apart at different sites within the Coastal savannah. The mean number of pathogens per animal as determined three months following the initial blood sampling from these herds from Shai Hills (40 cattle), LIPREC (46 cattle) and Ashiaman (41 cattle) were 1.2 ± 0.7, 1.6 ± 0.5 and 1.6 ± 0.7, respectively. These values increased progressively to 1.6 ± 0.4 (p = 0.001), 1.8 ± 0.5 (p = 0.03) and 2.0 ± 0.5 (p = 0.002) at the end of the 12-week study period, indicating ongoing transmission. These increases in the number of cattle acquiring additional pathogens were statistically significant in all three locations using a one-tailed *t*-test.

### Relationship of environmental variables to infection prevalence

We next determined if the environmental variables were predictive of prevalence for the targeted pathogens. For *A*. *marginale* prevalence, the best model included rainfall and EVI as significant predictors with an adjusted coefficient of determination (Adj. R^2^) of 0.805 (p = 2.22x10^-5^) and root mean square error (RMSE) of 0.086. For *B*. *bigemina* prevalence, the best model included rainfall, EVI and their interaction with an Adj. R^2^ = 0.586 (p = 0.005) and RMSE = 0.017. For *Theileria* spp. prevalence, the best model included only rainfall with an adjusted R^2^ = 0.727 (p = 3.30×10^-5^) and RMSE = 0.086 (Table 2). Besides the best models just described (Table 2), there was some, albeit weaker, support for competing models predicting each response (Table 3). For *A*. *marginale* prevalence, an evidence ratio of 3.27 indicated 3.27 times more support for the best model (AICc Wt = 0.71) than the second best model based on rainfall alone (AICc Wt = 0.22). The best model for *B*. *bigemina* also included not just the rainfall and EVI predictors, but also their interaction; this model (AICc Wt = 0.59) was stronger than the second best model without the interaction term (AICc Wt = 0.22) by an evidence ratio of 2.76. Finally, *Theileria* spp. prevalence was best predicted by rainfall alone (AICc Wt = 0.58), although there was weak yet equal support (AICc Wt = 0.21) for competing models that included maximum temperature, either with or without an interaction term (Table 3). The evidence ratio in support of the rainfall-only model was 2.80.

**Table 2:** Prevalence predicted by the best multiple linear regression models for each pathogen as determined by best subsets variable selection and minimum Akaike Information Criterion corrected (AICc) for small sample size.

Pathogen	Parameter	Estimate	Std. Error	t Value	Pr(>|t|)	Significance^a.^
*A*. *marginale*	Intercept	1.71	0.15	11.79	5.88 x10^-8^	***
	Rainfall	-2.81 x10^-3^	5.47 x10^-4^	-5.14	2.45 x10^-4^	***
	EVI	-8.33 x10^-5^	3.37 x10^-5^	-2.48	2.92 x10^-2^	*
	Model Fit Statistics:		RMSE^b.^ = 0.086	Adj. R^2^ = 0.805	p = 2.22 x10^-5^	***
*B*. *bigemina*	Intercept	-0.15	-0.11	-1.37	0.20	
	Rainfall	3.57 x10^-4^	3.50 x10^-4^	1.02	0.33	
	EVI	1.16 x10^-4^	4.10 x10^-5^	2.86	0.02	*
	Rainfall:EVI	-3.15 x10^-7^	1.27 x10^-7^	-2.49	0.03	*
	Model Fit Statistics:		RMSE^b.^ = 0.017	Adj. R^2^ = 0.586	p = 0.005	**
*Theileria* spp.	Intercept	1.03	0.14	7.48	4.63 x10^-6^	***
	Rainfall	-2.81 x10^-3^	4.54 x10^-4^	-6.18	3.30 x10^-5^	***
	Model Fit Statistics:		RMSE^b.^ = 0.086	Adj. R^2^ = 0.727	p = 3.30 x10^-5^	***

^a.^ *** p<0.001; ** p<0.01; * p<0.05

^b.^ RMSE = Root Mean Square Error.

**Table 3:** Model averaging based on corrected Akaike Information Criterion (AICc) weight (Wt).

Pathogen	Model^a.^	AICc^b.^	Delta AICc^c.^	AICc Wt^d.^	Cum. Wt^e.^
*A*. *marginale*	Rainfall + EVI	-19.19	0.00	0.71	0.71
	Rainfall	-16.82	2.37	0.22	0.93
	Rainfall + EVI + Rainfall:EVI	-14.53	4.66	0.07	1.00
	EVI	-5.56	13.63	0.00	1.00
*B*. *bigemina*	Rainfall + EVI + Rainfall:EVI	-62.75	0.00	0.59	0.59
	Rainfall + EVI	-60.72	2.03	0.22	0.81
	Rainfall	-60.39	2.36	0.18	0.99
	EVI	-54.46	8.29	0.01	1.00
*Theileria* spp.	Rainfall	-23.21	0.00	0.58	0.58
	Rainfall + MaxTemp + Rainfall:MaxTemp	-21.15	2.06	0.21	0.79
	Rainfall + MaxTemp	-21.15	2.06	0.21	1.00
	MaxTemp	-6.93	16.28	0.00	1.00

^a.^ Statistical models listed in order of strength of evidence supporting the model.

^b.^ AICc: A measure of the relative quality of competing statistical models, corrected for a small sample size (n = 15 sites).

^c.^ Delta AICc: The difference between the best model (smallest AICc, Table 2) and each subsequent model.

^d.^ AICc Wt: Weighted AICc, which is the probability that a particular model is the best model.

^e.^ Cum. Wt: Cumulative AICc Wt.

## Discussion

Ghana is a tropical country. However, as has been previously described and as reported here, there are significant regional differences in minimum and maximum temperature, relative humidity and rainfall as well as greater seasonal variation in these parameters in the Guinea savannah and the semi-deciduous forest zone relative to the coastal savannah [23]. The prevalence of the examined tick-borne pathogens was statistically significantly higher in the coastal savannah than in the semi-deciduous forest zone or the Guinea savannah. These findings support the hypothesis that within the context of a national tropical climate, there are significant regional differences in pathogen prevalence. We additionally demonstrated that rainfall and EVI, were highly predictive of pathogen prevalence in the three major livestock raising regions of Ghana. The most robust model was for *A*. *marginale* with rainfall and EVI together being the most predictive of prevalence. Similarly, rainfall and EVI together along with their interaction were most predictive of *B*. *bigemina* prevalence. Rainfall alone was most predictive for the prevalence of *Theileria* species. *A*. *marginale* and *B*. *bigemina* are generally transmitted by *Rhipicephalus* ticks, while *Theileria* spp. are more commonly transmitted by ticks within the genera *Hyalomma* and *Amblyomma* [17]. While the differences in optimum model parameters are modest, they may reflect differences in the preferred habitat and environment of these tick species.

Arthropod vectors have specific environmental requirements for survival and reproduction, such as temperature, humidity and host availability. Accordingly, the distribution and prevalence of vectors and thus vector-borne pathogens is more strongly linked to environmental variables than are other infectious diseases [24, 25]. Consequently, remote sensing, geographic information systems and predictive models based on vector populations are often used as indicators of disease risk [26, 27]. In particular, these models are likely to be robust in regions where a particular pathogen is transmitted by a single vector species and disease surveillance is relatively good. For example in the eastern U.S., *Borrelia burgdorferi*, the cause of Lyme disease, is maintained in a sylvatic cycle and spillover transmission to humans occurs solely when nymphal *Ixodes scapularis* feed on humans. The risk of Lyme disease is directly linked to the abundance of *I*. *scapularis* nymphs, which is absent in the winter and most abundant in early summer [28].

In tropical regions, these predictive modeling approaches can have major limitations [26, 29]. Model validation is predicated on the accurate determination of distribution and abundance of multiple tick species both spatially and temporally. However, vectors are difficult to detect within the environment, are not uniformly distributed among animals, and thus accurate and meaningful enumeration is difficult [30]. For tick populations, this is particularly true in tropical regions which are often less accessible and where multiple tick vector species are present year around, but can vary in abundance seasonally and by life stage [30, 31]. For example, more than nine different tick species, many of which serve as vectors for more than one pathogen, have been identified on cattle in the livestock rearing vegetation zones in Ghana [30, 32]. The competence and vector capacity of each of these ticks and their life stages and the degree to which each contributes to the overall pathogen burden is unknown. Thus risk assessment for many vector-borne diseases, particularly within the tropics, must be based on parameters that predict disease incidence and prevalence rather than solely on the abundance and distribution of a vector species. Readily obtainable environmental data such as rainfall and EVI can be used to predict pathogen prevalence, as demonstrated by the data presented here.

The spatial (Ghana) and temporal (three years) ranges of the data considered in our study were too limited to detect or infer effects of global warming. In this context, however, regional shifts in climate may have the largest impact in resource poor regions and the ability to accurately predict the effect of such alterations allows for targeting limited resources for long term planning and mitigation of negative outcomes. Livestock populations at greatest risk for increased morbidity and mortality from tick borne pathogens are those that initially have lower prevalence of infection and thus less herd immunity and then experience a climate-based shift to more intense tick transmission. In addition, increased seasonal or interannual variation may result in periods of low tick transmission leaving increasingly higher numbers of susceptible animals in a herd. Episodic periods of high transmission could then lead to periodic disease outbreaks characterized by high morbidity and mortality.

Greater spatial resolution of rainfall data and disease incidence than that reported in this paper may allow for finer resolution mapping and increased accuracy of predicting how altered climatic variables and closely coupled vegetation dynamics affect pathogen transmission. Resolution of such predictions on the scale of administrative districts is essential for efficient targeting of limited resources for mitigation efforts within a country. The current data suggest that relatively small shifts in regional rainfall and vegetation may result in significant shifts in pathogen prevalence. Though the ecology of different vector borne pathogens will vary, these data demonstrate that readily available weather and satellite data can be used to predict pathogen prevalence and thus ultimately disease risk.
